# Bradykinin promotes immune responses in differentiated embryonic neurospheres carrying APP^swe^ and PS1^dE9^ mutations

**DOI:** 10.1186/s13578-024-01251-3

**Published:** 2024-06-18

**Authors:** Guilherme Juvenal, Carine Meinerz, Ana Carolina Ayupe, Henrique Correia Campos, Eduardo Moraes Reis, Beatriz Monteiro Longo, Micheli Mainardi Pillat, Henning Ulrich

**Affiliations:** 1 Department of Biochemistry, Institute of Chemistry, University of São Paulo, Av. Prof. Lineu Prestes 748, São Paulo, 05508-900 Brazil; 2 Department of Microbiology and Parasitology, Health Sciences Center, Federal University of Santa Maria, Santa Maria-RS, Brazil; 3 Department of Physiology, University of São Paulo, São Paulo, SP Brazil

**Keywords:** Alzheimer’s disease, Immune response, Stem cells, Gene expression, Biological system

## Abstract

**Background:**

Neural progenitor cells (NPCs) can be cultivated from developing brains, reproducing many of the processes that occur during neural development. They can be isolated from a variety of animal models, such as transgenic mice carrying mutations in amyloid precursor protein (APP) and presenilin 1 and 2 (PSEN 1 and 2), characteristic of familial Alzheimer’s disease (fAD). Modulating the development of these cells with inflammation-related peptides, such as bradykinin (BK) and its antagonist HOE-140, enables the understanding of the impact of such molecules in a relevant AD model.

**Results:**

We performed a global gene expression analysis on transgenic neurospheres treated with BK and HOE-140. To validate the microarray data, quantitative real-time reverse-transcription polymerase chain reaction (RT-PCR) was performed on 8 important genes related to the immune response in AD such as CCL12, CCL5, CCL3, C3, CX3CR1, TLR2 and TNF alpha and Iba-1. Furthermore, comparative analysis of the transcriptional profiles was performed between treatments, including gene ontology and reactome enrichment, construction and analysis of protein-protein interaction networks and, finally, comparison of our data with human dataset from AD patients. The treatments affected the expression levels of genes mainly related to microglia-mediated neuroinflammatory responses, with BK promoting an increase in the expression of genes that enrich processes, biological pathways, and cellular components related to immune dysfunction, neurodegeneration and cell cycle. B2 receptor inhibition by HOE-140 resulted in the reduction of AD-related anomalies caused in this system.

**Conclusions:**

BK is an important immunomodulatory agent and enhances the immunological changes identified in transgenic neurospheres carrying the genetic load of AD. Bradykinin treatments modulate the expression rates of genes related to microglia-mediated neuroinflammation. Inhibiting bradykinin activity in Alzheimer’s disease may slow disease progression.

**Supplementary Information:**

The online version contains supplementary material available at 10.1186/s13578-024-01251-3.

## Backgound

Neural progenitor cells (NPCs) are present during embryonic brain development and in the subventricular zone and dentate gyrus of the adult central nervous system (CNS). Adequate in vitro culture of NPCs from rat and mouse allows their proliferation and aggregation originating neurospheres [1, 2]. Neurosphere differentiation encompasses many of the complex processes that occur during early stages of neural development, such as proliferation, migration, neurogenesis and gliogenesis [3, 4]. Furthermore, the possibility of isolating and studying NPCs from a variety of animal models, such as transgenic and knockout mice for disease modeling, makes these cells a very effective system [5].

In this context, the use of neurospheres is a tool to study pathological mechanisms of neurodegenerative diseases, such as Alzheimer’s disease (AD). In familial AD (fAD), mutations in amyloid precursor protein (APP) and presenilin 1 and 2 (PSEN 1 and 2) promote disrupted neurogenesis and reduced learning and memory in animal models, such as double transgenic mice expressing a chimeric mouse/human amyloid precursor protein (Mo/HuAPP695swe) and a mutant human presenilin 1 (PS1-dE9) (APP/PS1), both directed to CNS neurons [6]. The presence of this genetic load has been reproduced in APP/PS1 neurospheres differentiation. In a recent study of our group using APP/PS1 neurospheres, we demonstrated that several features observed in the adult brain with AD, such as innate immunity and inflammation induction, are spontaneously present, suggesting an early neurodevelopmental origin for familial AD [7]. In this previous work, we showed that differentiated APP/PS1 neurospheres exhibit up to 100-fold higher expression rates of C-C motif chemokine 12, C-C motif chemokine 5, C-C motif chemokine 3, Complement C3, CX3C chemokine receptor 1, Toll-like receptor 2 and tumor necrosis factor alpha (CCL12, CCL5, CCL3, C3, CX3CR1, TLR2 and TNF alpha, respectively). Iba-1 (allograft inflammatory factor 1) was 20-fold upregulated, indicating the presence of activated microglia in APP/PS1 neurospheres. The secretome of neural-differentiated APP/PS1 NPCs showed enhanced chemoattraction of PBMCs, validating the APP/PS1 differentiated neurospheres as an in vitro model to study developmental origins of familial AD [7].

One of the main features of the progression of AD is neuroinflammation [8], which is characterized by the activation of glial cells and the production of inflammatory factors [9]. Among all inflammatory modulators, several peptides act in the CNS, including bradykinin (BK). The kinins BK and kallidin (Lys^10^-BK) are oligopeptides formed by the cleavage of kininogens by kallikreins. Their biological effects are caused by the activation of two transmembrane receptors, kinin-B1 and B2 receptors. BK has a high affinity for the B2 receptor, which is constitutively expressed in many cells, including microglia [10–14], and is involved in inflammatory processes. In addition, elevated plasma levels of BK have been associated with memory loss in AD [15].

To investigate the effects of BK in AD, we conducted a global transcriptional analysis of APP/PS1 neurospheres, which had been induced to neural differentiation in the presence BK or the B2 receptor antagonist HOE-140. Considering the roles of the kinin system in neuroinflammation, we hypothesized that treatment with BK intensifies AD-related immune system activation, while HOE-140 treatment mitigates these effects. We observed that the treatments affected the expression of genes mainly related to microglia-mediated neuroinflammation, with BK promoting an increase in the expression of genes that enrich processes, biological pathways and cellular components related to immune system dysfunction, neurodegeneration and cell cycle control.

## Methods

### Neurosphere formation assay

B6.Cg-Tg (APPswe, PSEN1dE9, 85Dbo/J) transgenic mice, acquired from Jackson Laboratory (JAX® Mice and Services, Bar Harbor, Maine, MA), were used for NPC isolation and neurosphere generation. Mice were genotyped by using the polymerase chain reaction (PCR) (see Supplementary Table S1 for primer sequences). This work was approved by the Ethics’ Committee of the Universidade Federal de São Paulo (UNIFESP) under the authorization number CEUA 1,435,020,517.

Timed-pregnant wild-type or APP/PS1 C57BL/6 mice were obtained by overnight mating. On day thirteen of gestation, these females were sacrificed, and the telencephalons of their embryos (E13) were isolated and then mechanically and enzymatically dissociated [16, 17]. Cells were grown in suspension at a density of 2 × 10^5^ cells/ml in 2% B-27 (Life Technologies, Carlsbad, CA) Dulbecco’s modified Eagle’s medium (DMEM) with Ham’s F-12, antibiotics (100 units/ml penicillin and 100 g/ml streptomycin), 20 ng/ml pro-epidermal growth factor (EGF) and 20 ng/ml fibroblast growth factor 2 (FGF2) (Sigma Aldrich, St Louis, MO) and maintained at 37 °C in a water-saturated atmosphere with 5% of CO_2_. After six days of incubation, neurospheres were generated. For differentiation, neurospheres were washed twice with DMEM/F12 medium following centrifugation for 3 min at 100xg and then were allowed to attach to poly-L-lysine and laminin precoated plastic flasks and cultured with DMEM/F-12, supplemented with 2% B-27, in the absence of FGF-2 and EGF. Neural progenitor cells were differentiated for 7 days in the incubator at 37 °C with controlled humidity and 5% CO_2_.

### Drug Treatment

Neurospheres from APP/PS1 (APP_WT) mice were submitted to different drug treatments on days 0, 3, and 6 of the differentiation phases. APP_WT neurospheres were maintained untreated; BK_APP neurospheres were treated with 1µM BK (Tocris Bioscience) and HOE_APP neurospheres were treated with 1µM HOE-140 (Tocris Bioscience). Drug concentrations were defined on Pillat et al. 2016 [16]. On the seventh day of differentiation, RNAs were extracted from neurospheres in order to perform RT-real time PCR and microarray analysis.

### RNA extraction

Total RNAs of WT, APP_WT, BK_APP, and HOE_APP neurospheres were extracted using the RNAspin Mini Kit (GE Healthcare, Chalfont St Giles, UK), according to the manufacturer’s instructions. Isolated RNAs were treated with DNAse. Their concentrations and qualities were verified by UV spectrophotometry and agarose gel electrophoresis, respectively.

### Gene expression profiling with microarray analyses

Total RNA profiles of neurospheres were analyzed using the Agilent SurePrint G3 Mouse GE 8 × 60 K Microarray slides (Agilent Technologies, Santa Clara, CA). The following comparisons of gene expression profiles were performed: APP_WT *versus* WT neurospheres; HOE_APP *versus* APP_WT; and BK_APP *versus* APP_WT. For that, two-color array measurements with dye swapping using four independent biological replicates of each neurosphere model were elaborated. Cy3- and Cy5-labeled cRNA targets were generated by in vitro transcription using 100 ng total RNA from each neurosphere model using the Low Input Quick Amp Labeling Kit (Agilent Technologies) and incubated with the arrays using the Gene Expression Hybridization Kit (Agilent Technologies), as recommended by the manufacturer. The arrays were then washed as described on Agilent SSPE wash protocol v. 2.1 and scanned with a High-Resolution Microarray Scanner (Agilent Technologies). The Agilent Feature Extraction Software (version 9.5) was used to quantify the images. Probes that presented higher gene expression levels compared to corresponding background levels in 3 of the 4 replicates were considered as expressed and further analyzed. Two methods were selected to identify genes differentially expressed between neurosphere models: the significance analysis of microarray (SAM) approachs [18] and rank products [19]. Only genes classified as differentially expressed with *p* ≤ 0.05 by the two methods were considered for further analysis. Raw and normalized expression data were deposited in the GEO database (https://www.ncbi.nlm.nih.gov/geo) under accession GSE246792.

### Reverse-transcription and quantitative polymerase chain reaction

To validate the microarray data, quantitative real-time reverse-transcription polymerase chain reaction (RT-real time PCR) was performed on 8 genes related to the immune response in AD. Each reaction was performed with the same RNA samples tested in the microarray experiments. OligodT-primed reverse transcription (RT) was performed using 350 ng of total RNA according to the Super Script III kit protocol (Invitrogen). Relative transcript levels were determined by performing RT-PCR (the corresponding gene-specific primer pairs are listed in Table S1) with Power SYBR Green (Applied Biosystems) according to the delta Ct method [20] using a 7500 Real-Time PCR System (Applied Biosystems). GAPDH abundance levels were used to normalize the assay.

The student’s t-test implemented in the GraphPad Prism software (GraphPad Software Inc., version 3.00, CA) was used to analyze RT-real time PCR results. Differences in expression levels between strains were considered statistically significant when a *p* ≤ 0.05. Data are expressed as the mean ± standard error of the mean (SEM).

### Flow cytometry

CD11b-positive cells were evaluated as previously described [16]. Differentiated cells from WT and AD neurospheres were dissociated to a single cell suspension and were fixed with 2% PFA for 10 min. Samples were washed with PBS supplemented with 2% FBS and incubated for 30 min with a phycoerythrin-coupled antibody against CD11b PE (1:200 Sigma-Aldrich). Following a washing step with PBS, cells were analyzed on a Calibur flow cytometer (BD Biosciences). The argon laser line at 488 nM was used for fluorescence excitation and fluorescence emission was collected at 575 nm (FL2 peak fluorescence emission at 575 nm, collected with a band pass filter). Fifty-thousand events were acquired per sample and were analyzed with Flowjo V10. The results are reported as mean ± SEM of positively stained cells. The data shown are representative of at least three independent experiments. ** *p* < 0.01.

### Gene ontology and reactome enrichment

The human orthologous genes identified in the microarray for each group (HOE_APP, APP_WT, and BK_APP) and their respective fold changes (FCs) between treated and untreated samples were used to search for enriched pathways via Reactome version V85 and biological processes (BPs) and cellular components (CCs) via Gene Ontology (GO) annotations (version 2023-07-27). Only pathways with a False Discovery Rate (FDR) < 0.05 and BPs and CCs with a *p* < 0.05 were considered significantly enriched. The average fold change for each pathway, BP, or CC is calculated as the average FCs of the genes enriching the annotation.

### Correlation of gene expression between treatments

Using the microarray expression measurements described above, a new FC calculation was made to compare APP/PS1 treated groups to the WT control, thus generating two new groups: HOE_WT and BK_WT. The putative FCs for each gene contained in HOE_WT is the sum of the FCs of the gene in APP_WT and the FCs in HOE_APP. For the BK_WT group, the putative FCs is the sum of the FCs in APP_WT and in BK_APP. With the FCs of APP_WT and the putative FCs of HOE_WT and BK_WT, Pearson correlations (PC) were calculated for each gene considering the order HOE_WT, APP_WT, and BK_WT as a hypothesis of increasing progression of immune impairment. Only genes with absolute PC values greater than 0.9 and *p* < 0.05 were further evaluated.

### Construction of protein-protein interaction networks and calculation of weighted system impact (wSI)

For each set of identified human orthologous coding genes, identified by Ensembl (version 110), a protein-protein interaction (PPI) network was constructed. Firstly, the set was submitted to UniProt version 2023_04 to search for interactions and interacting proteins. Then each interaction was weighted with the inverse of the median between the FCs of the genes participating. Finally, the networks were built using an algorithm in Python. The closeness and betweenness were calculated for each gene, and the sum between these two values was called weighted system impact (*wSI*). The metric proposed here has the function of indicating the “dominance” of a protein over a PPI network.

### Comparison with human dataset

Comparisons were made by using a tissue dataset from AD patients available in the NCBI GEO database with code GSE118553. The dataset includes the transcriptome of tissue fragments from the entorhinal cortex (EC), temporal cortex (TC), frontal cortex (FC), and cerebellum (CB) of 112 patients. The samples were subdivided into three groups: twenty-seven samples in control cases, classified as showing no clinical sign of any form of dementia and no neuropathological evidence of neurodegeneration; thirty-three samples, defined as clinically dementia-free at the time of death, but neuropathological assessment at autopsy showing hallmark AD pathology (AsymAD) and fifty-two samples of AD patients, who had clinical diagnosis of AD at death and confirmation of this diagnosis through neuropathological evaluation at autopsy [21]. PPI networks were constructed for each tissue and condition of AD patients. The isolated sets were deleted and only the main set was used in this analysis. The FCs and wSI of the genes identified in patients and neurosphere samples were used to construct Pearson correlation matrices and heatmaps with hierarchical clustering employing the Ward method with the Euclidean distance.

## Results

### Gene expression profiling in differentiated embryonic neurospheres

**Fig. 1:**
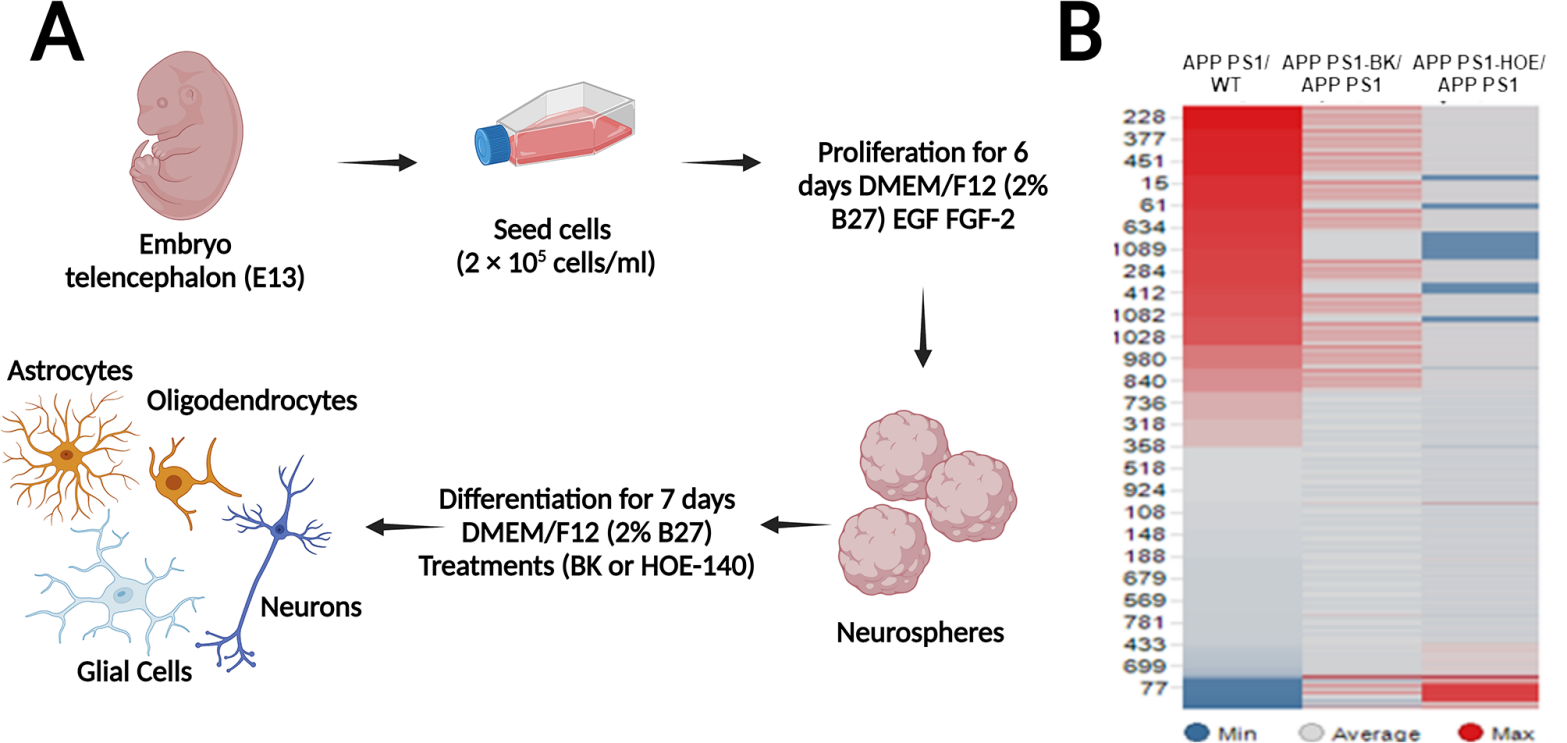
Workflow of neurosphere preparation and differentiation, and heatmap of gene relative expression. **A** Schematic representation of fundamental steps for performing neurosphere assays obtained from the telencephalon of mouse embryos (E13). **B** Altered gene expression profiles comparing APP_WT with WT neurospheres; BK_APP with APP_WT neurospheres and HOE_APP with APP_WT neurospheres. Heatmaps showing the expression ratios (log_2_) of genes related to the immune response of four independent replicates (R1-R4) of the comparison described above. Differentially expressed transcripts were identified using SAM and rank products (*p* ≤ 0.05)

We used microarray technology to study the genome-wide gene expression profiles in differentiated NPCs, obtained from embryo telencephalons (E13; Fig. 1A). Neurospheres were obtained from WT and APP^swe^/PS1^dE9^ mice. The transgenic neurospheres were divided in three groups: untreated (APP_WT), treated with BK (BK_APP), and treated with HOE-140 (HOE_APP). The different neurosphere models presented a total of 3,639; 978 and 835 (*p* < 0.05) transcripts with differences in gene expression between neurospheres WT and APP_WT, HOE_APP and APP_WT and BK_APP and APP_WT, respectively.

Our results showed a specific enrichment in gene expression of APP_WT neurospheres, especially for genes related to immune responses (first column of Fig. 1B). This result corroborates the data published in our previous paper [7]. Controls used for this analysis are the same ones that allowed us to obtain consistent data in our previous work, and were reanalyzed for the data reported here.

In our current study, we found that BK treatment (BK_APP neurospheres) increased the expression of genes related to immune responses (Fig. 1B, second column). Conversely, expression levels of these genes was decreased in HOE_APP neurospheres (Fig. 1B, third column).

### Expression changes in neurospheres treated with BK and HOE-140

**Fig. 2:**
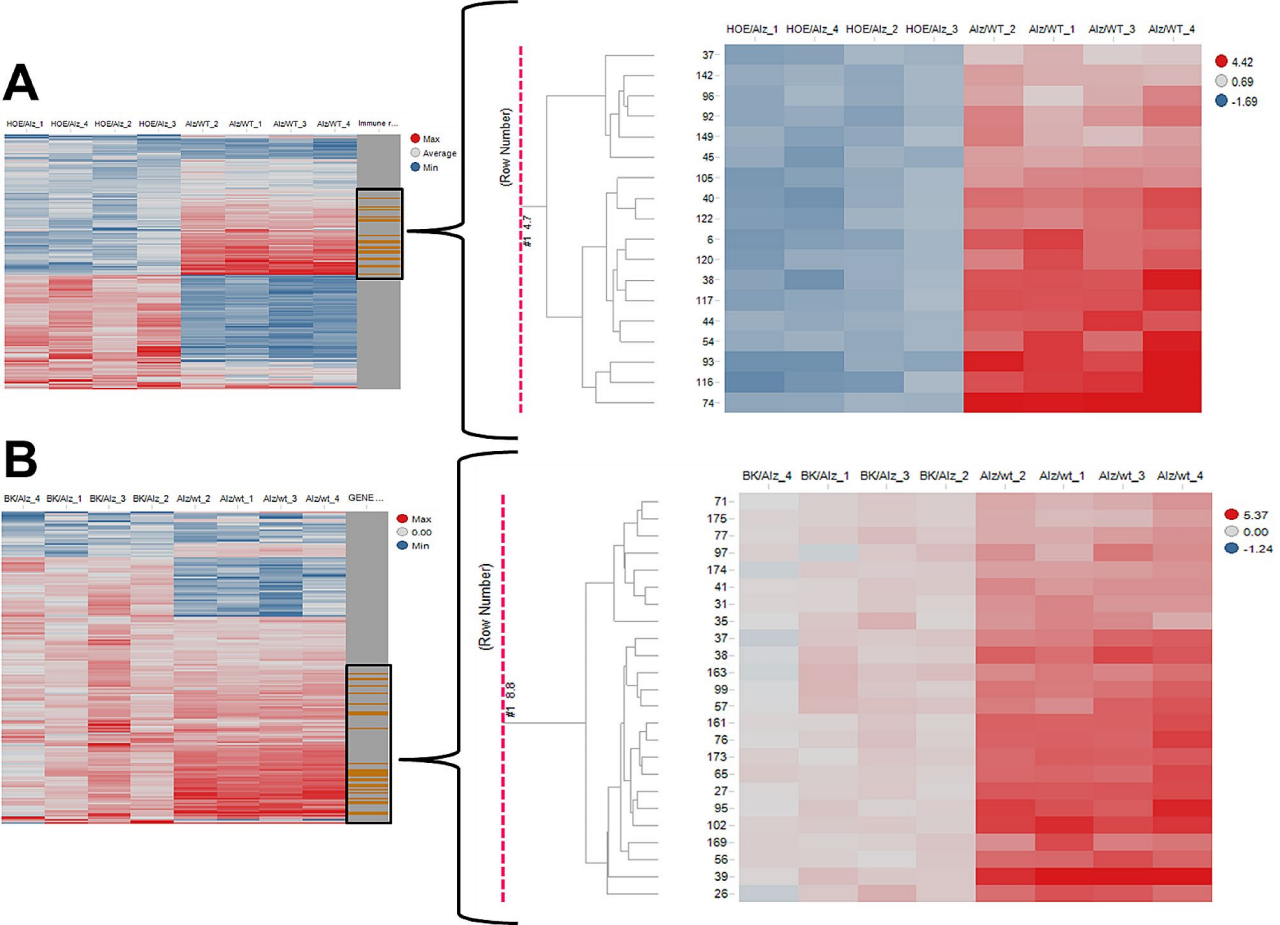
Gene expression changes in transgenic neurospheres treated with BK and HOE-140. **A** Heatmap with emphasis on genes related to immune responses, which were underexpressed in HOE_APP neurospheres. **B** Heatmap with emphasis on genes related to immune responses, which were overexpressed in BK_APP neurospheres

To understand the effects of BK and HOE-140 treatments on differentiated AD neurospheres, we determined the genes, which were significantly enriched in pathways related to immune responses. Comparison between the expression in APP_WT and HOE_APP genes demonstrated 18 genes, which were underexpressed: bone marrow stromal antigen 2, complement C4-B, C-C motif chemokine 5, CX3C chemokine receptor 1, C-X-C motif chemokine 10, antiviral innate immune response receptor RIG-I, guanylate-binding protein 5, guanylate-binding protein 6, psychosine receptor, interferon-induced helicase C domain-containing protein 1, interferon-induced protein with tetratricopeptide repeats 1, interferon-induced protein with tetratricopeptide repeats 3, ubiquitin-like protein ISG15, lymphocyte antigen 86, tyrosine-protein phosphatase non-receptor type 6, S-adenosylmethionine-dependent nucleotide dehydratase RSAD2, Toll-like receptor 2 and zinc finger CCCH-type antiviral protein 1 (BST2, C4B, CCL5, CX3CR1, CXCL10, DDX58, GBP5, GBP6, GPR65, IFIH1, IFIT1, IFIT3, ISG15, LY86, PTPN6, RSAD2, TLR2 and ZC3HAV1, respectively) in neurospheres treated with HOE-140 (Fig. 2A). Twenty-four genes were overexpressed in BK_APP compared to APP_WT: B-cell lymphoma 3 protein, B-cell linker protein, complement C1q subcomponent subunit B, caspase-4, C-C motif chemokine 2, C-C motif chemokine 3, C-C motif chemokine 4, monocyte differentiation antigen CD14, CX3C chemokine receptor 1, C-X-C motif chemokine 5, adhesion G protein-coupled receptor E1, fatty acid synthase, guanylate-binding protein 5, GTP cyclohydrolase 1, interferon-induced protein with tetratricopeptide repeats 1, immunoglobulin superfamily member 6, ubiquitin-like protein ISG15, leukocyte immunoglobulin-like receptor subfamily B member 4, Toll-like receptor 2, tumor necrosis factor alpha, triggering receptor expressed on myeloid cells 2, TYRO protein tyrosine kinase-binding protein, proto-oncogene vav and vascular cell adhesion protein 1 (BCL3, BLNK, C1QB, CASP4, CCL2, CCL3, CCL4, CD14, CX3CR1, CXCL5, EMR1, FAS, GBP5, GCH1, IFIT1, IGSF6, ISG15, LILRB4, TLR2, TNF-alpha, TREM2, TYROBP, VAV1 and VCAM1, respectively) in BK-treated neurospheres (Fig. 2B).

Among the 37 genes modulated by the treatments, five showed opposite behaviors, being overexpressed in conditions of BK stimulation and underexpressed when the B2 receptor had been blocked with HOE-140: CX3CR1, GBP5, IFIT1, ISG15, and TLR2.

### Quantitative expression analysis of genes of differentiated APP^swe^/PS1^dE9^ neurospheres

The expression of eight important genes linked to inflammatory responses and cell-to-cell communication in the CNS was investigated by RT-real time PCR. In a previous study of our group [7], the expression of these genes was significantly increased in APP/PS1neurospheres, and it was now our interest to evaluate the expression pattern of these genes in BK /HOE-140 treated neurospheres.

**Fig. 3:**
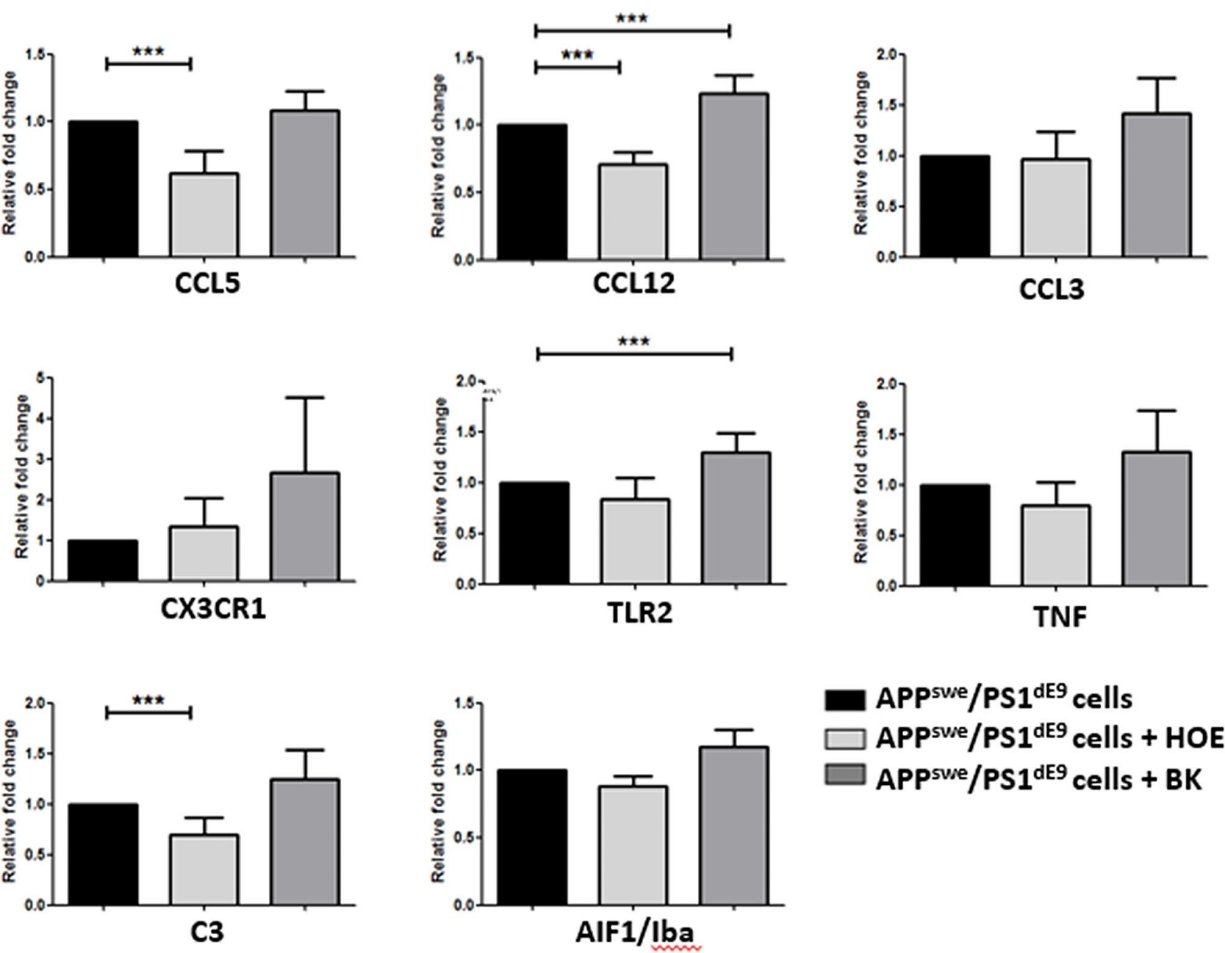
mRNA expression levels of 8 inflammatory genes in neurospheres. The experiments were performed by RT-real time PCR using at least four independent replicates. GAPDH gene expression was used to normalize expression levels (Paired t-test, two-tailed; ***p ˂0.05)

The results (Fig. 3) revealed a significant decrease (*p* < 0.05) in C3, CCL12, and CCL5 expression in HOE_APP neurospheres compared to APP_WT neurospheres. Expression of TLR2 and CCL12 was significantly increased (*p* < 0.05) in BK_APP neurospheres compared to APP_WT neurospheres. There were no significant differences in the expression of CCL3, CX3CR1, TNF, and Iba-1 genes between treated and untreated neurospheres.

The presence of microglia in neurospheres is expected since these cells are known to migrate to the brain around the ninth day of embryonic development. Ginhoux and colleagues (2013) described that, with the establishment of the circulatory system, which occurs from E8.5 to E10, yolk-sac derived primitive macrophages would spread into the embryo through the blood, migrating to various tissues, including the brain, where they proliferate and colonize [23]. Since NPCs were isolated from embryo telencephalon (day 13; E13) of wild-type (WT) or APPswe/PSldE9 C57BL/6 mice embryos, macrophages already have migrated to the brain, where they differentiate into microglia. The RNA-seq analysis further corroborated the infiltration hypothesis. Microglial markers, including Aif1, Fcrls, Hexb, and Tmem119, were upregulated in their expression levels in APP/PS1 compared to WT neurospheres [7]. The Aif1 gene, that encodes the Iba protein, is also identified in the BK_APP. Expression of this microglial marker had a tendency of upregulation following BK treatment (Fig. 3). Additionally, samples from AD-differentiated neurospheres exhibited a higher frequency of CD11b-positive cells compared to those from WT animals, indicating the presence of activated microglia in this AD model (Fig. S1).

### Reactome enrichment

**Fig. 4:**
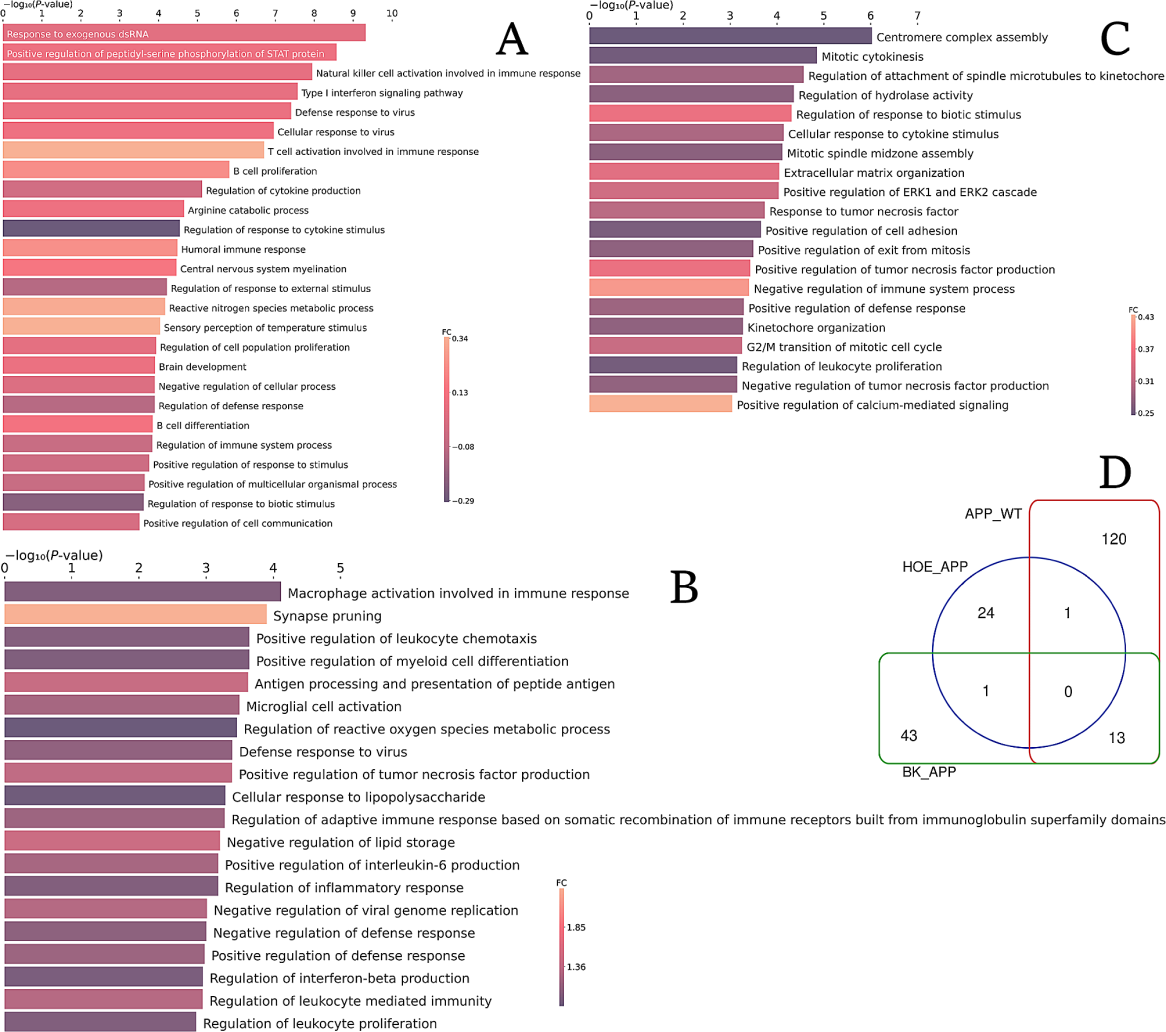
BPs by GO enrichment and their respective average FCs. **A, B**, and **C**, HOE_APP, APP_WT, and BK_APP genes, respectively. In the last two groups, only the 20 BPs with the biggest FCs are shown. In **D** the number of common processes between the groups is presented

**Fig. 5:**
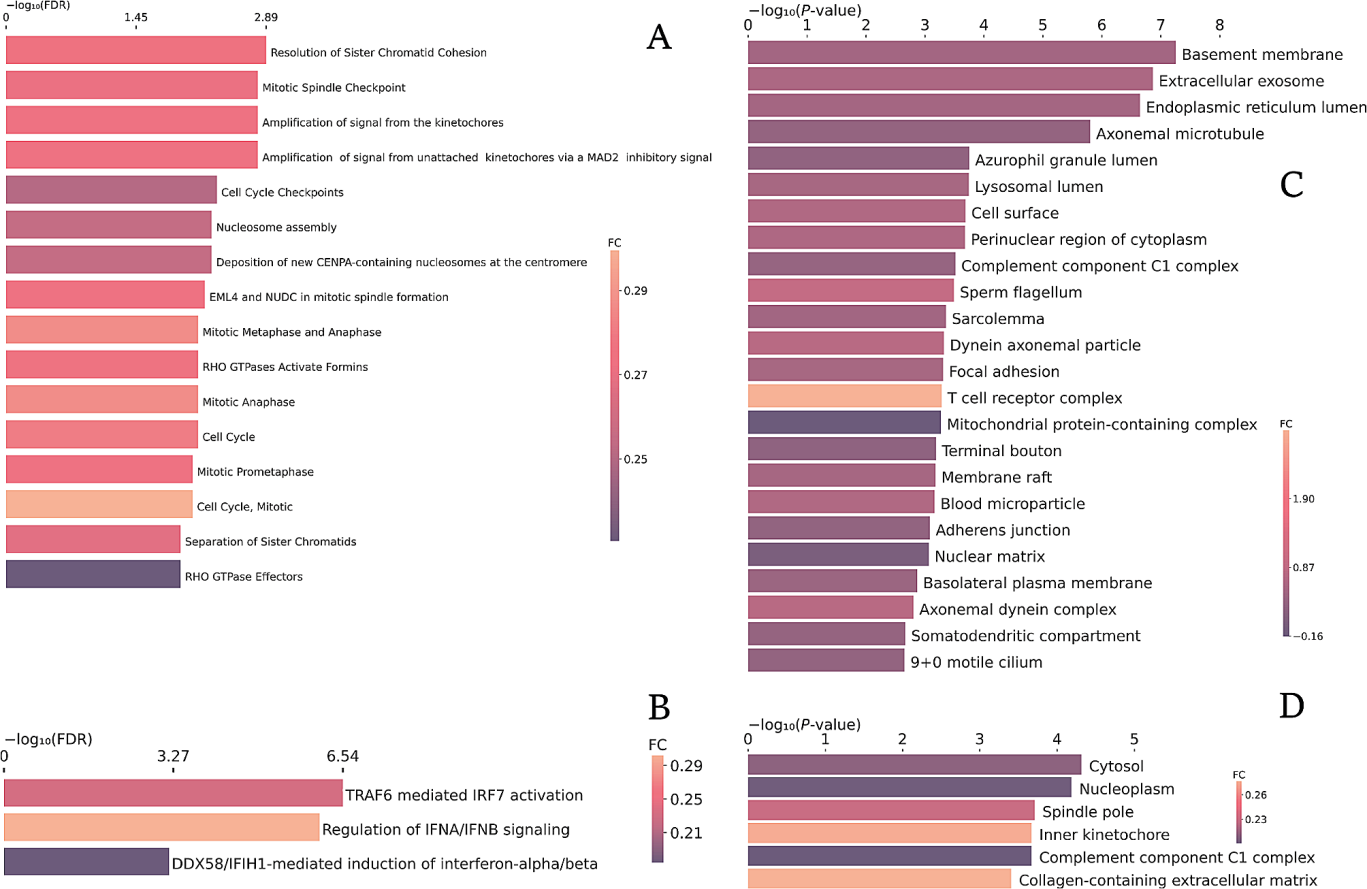
Enrichment of pathways by Reactome and CCs by GO and their respective average FCs. **A** and **B** pathways enriched in Reactome with all genes identified in BK_APP and HOE_APP, respectively; **C** and **D**, GO-enriched CCs with all genes identified in APP_WT and BK_APP, respectively

The human orthologous genes assigned to mouse genes detected in the microarrays and their respective FCs were used to analyze enriched pathways from Reactome and BPs and CCs from GO (Figs. 4 and 5). In GO, APP_WT displayed enrichment of 24 CCs and 135 BPs terms, HOE_APP genes were enriched in 26 BPs and BK_APP genes were enriched in 6 CCs and 57 BPs terms, respectively. No BP was found in common among the three groups (Fig. 4D). A similar analysis was performed for the enrichment of pathways in Reactome and CC in GO (Fig. 5). Only genes present in HOE_APP, and BK_APP groups generated significant enrichment of Reactome terms, with 3 and 16  enriched pathways, respectively.

### Correlation of gene expression between treatments

**Fig. 6:**
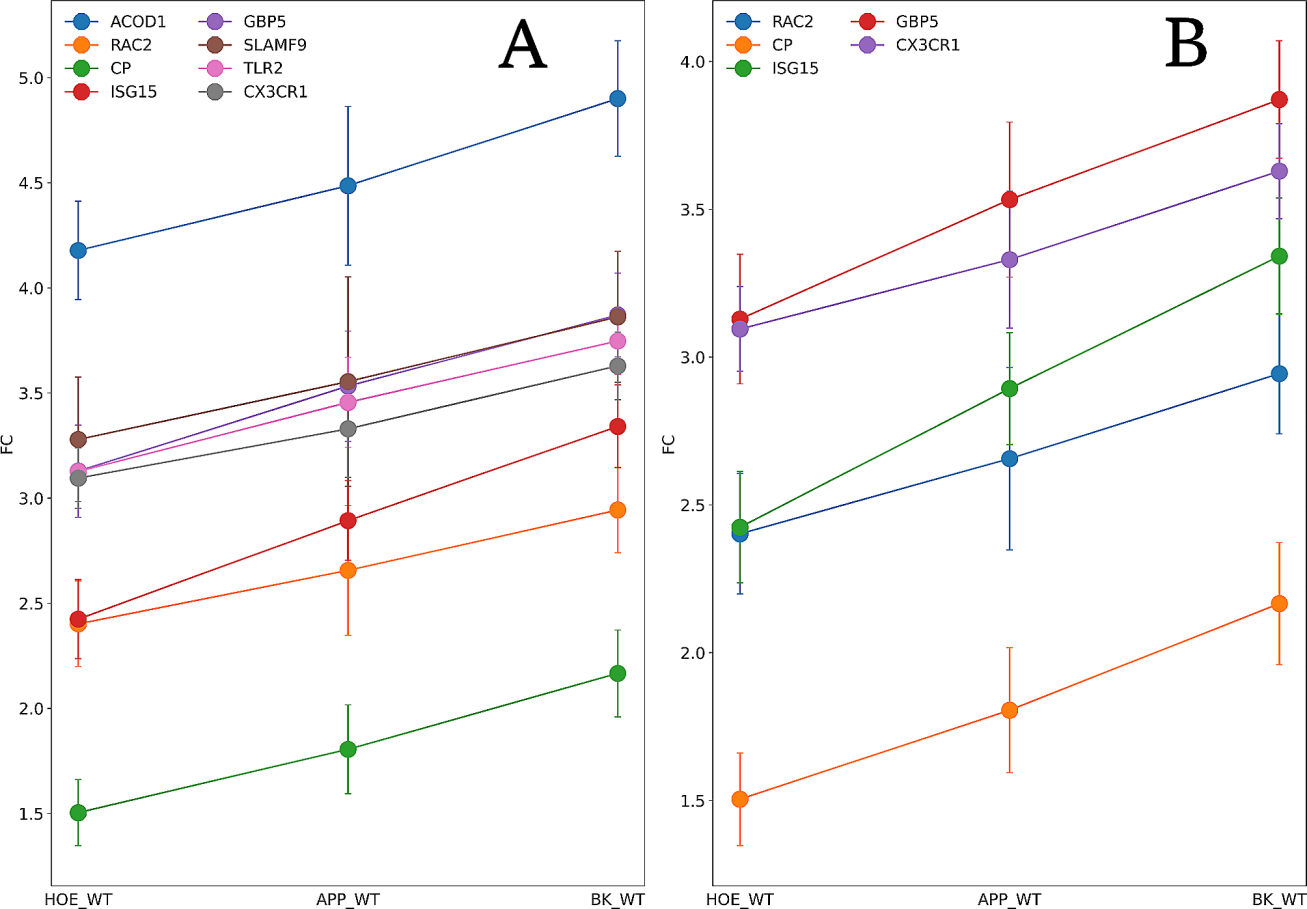
Proteins with an absolute Pearson’s correlation greater than 0.9 and a p-value lower than 0.05. **A** are arranged mouse proteins and in **B** their human orthologs

Based on our hypothesis, it was possible to propose that HOE_WT, APP_WT, and BK_WT is the order that characterizes the increasing impairment of the immune system. To investigate this proposal, we verified which genes vary their expression following this order, with absolute Pearson’s correlation > 0.9 and *p* < 0.05, (Fig. 6). The calculation of FCs referring to WT is only possible among the genes identified in the three groups (HOE_APP, APP_WT, and BK_APP), therefore, this analysis was performed with 25 mouse genes and 17 human orthologous genes. Eight mouse genes and five human orthologs genes fit this restriction, all with positive correlations (Fig. 6A, and B, respectively).

### Construction of protein-protein interaction (PPI) networks and calculation of weighted system impact (wSI)

**Fig. 7:**
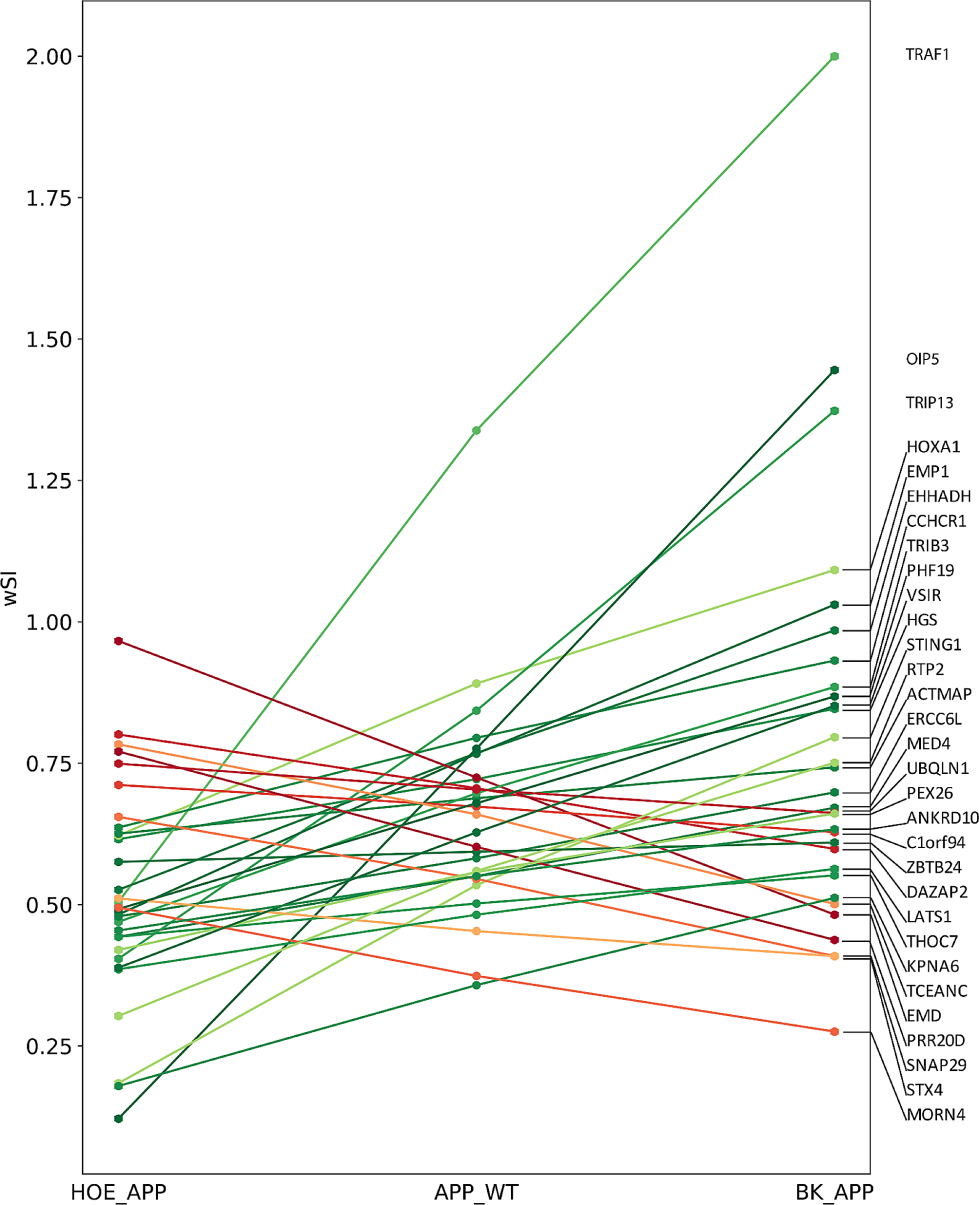
Proteins and *wSI*, which have absolute Pearson’s correlation greater than 0.9. Lines in shades of red represent progression with negative PC and those in shades of green with positive PC (p-value < 0.05)

The number of proteins and interactions in each PPI network are presented in (Fig. S2). The PC analysis in the HOE_APP, APP_WT, and BK_APP progression can also be done with the protein’s *wSI*, in order to highlight the role of treatments in the topological position of the proteins in the network (Fig. 7). Epithelial membrane protein 1, TNF receptor-associated factor 1, stimulator of interferon genes protein, synaptosomal-associated protein 29, syntaxin-4 and ubiquilin-1 (EMP1, TRAF1, STING1, SNAP29, STX4, and UBQLN1, respectively) in addition to having extreme PC values, are correlated with the immune system, with the first three being PC positive and the others being negative.

### Comparison with the transcriptome of human AD patients

In order to make a parallel between the findings determined through the murine neurosphere models and human patients, comparisons were made with a dataset of tissue from cases with Alzheimer’s available in the GEO database (GSE118553) [21]. The detected coding genes and their respective FCs between AD patients and normal controls were used for comparison with the transcriptomes of the neurosphere model and their treatments, in two ways: by FCs and *wSI* of the common genes between the groups. For these, PPI networks were constructed for each tissue and condition of patients with AD. Isolated sets were deleted and only the main set was used in this analysis.

**Fig. 8:**
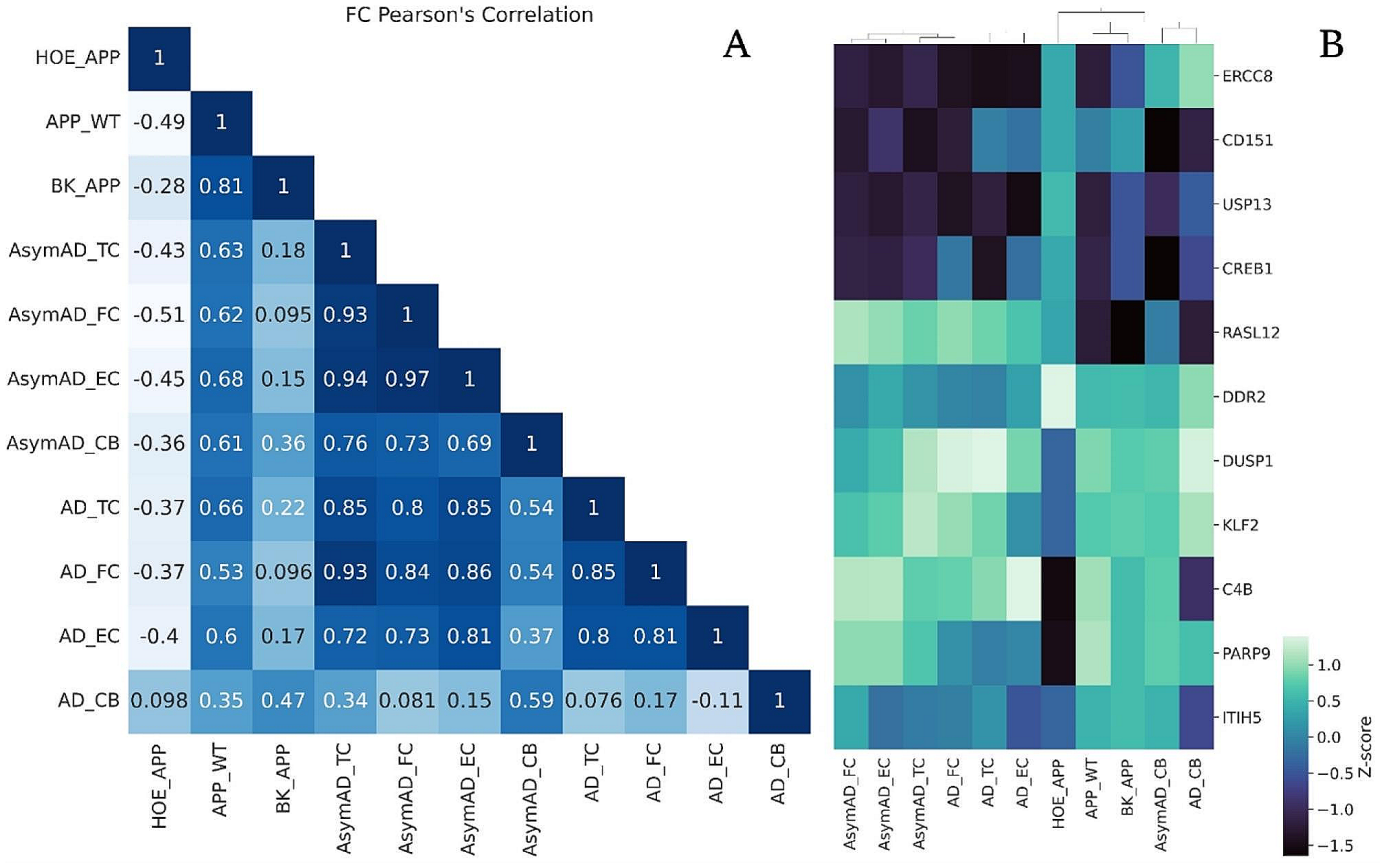
The 11 genes were detected in the microarray analysis and in the human dataset. **A** correlation between FCs of genes. **B** heatmap with hierarchical clustering of FCs of the 11 genes commonly detected in the murine neurospheres and AD patients’ tissues

The reorganization of the gene expression pattern generated by the treatments with HOE-140 and BK can be observed by the correlation of gene expression with the human profile (Fig. 8 and clusters in Table S2). Only 11 genes are shared between the neurosphere experimental groups and those obtained from the human dataset. The Pearson’s correlation values of these genes are moderately negative between the HOE_APP group and all regions of the human brain studied, except for the cerebellum, which has a low positive correlation. On the other hand, the APP_WT group mainly presents moderate positive correlations, and the BK_APP group has low positive correlations. This is an indication that treatment with HOE-140 generates a considerable change in the expression of these 11 transcripts compared to the expression in brain regions that AD compromises.

**Fig. 9:**
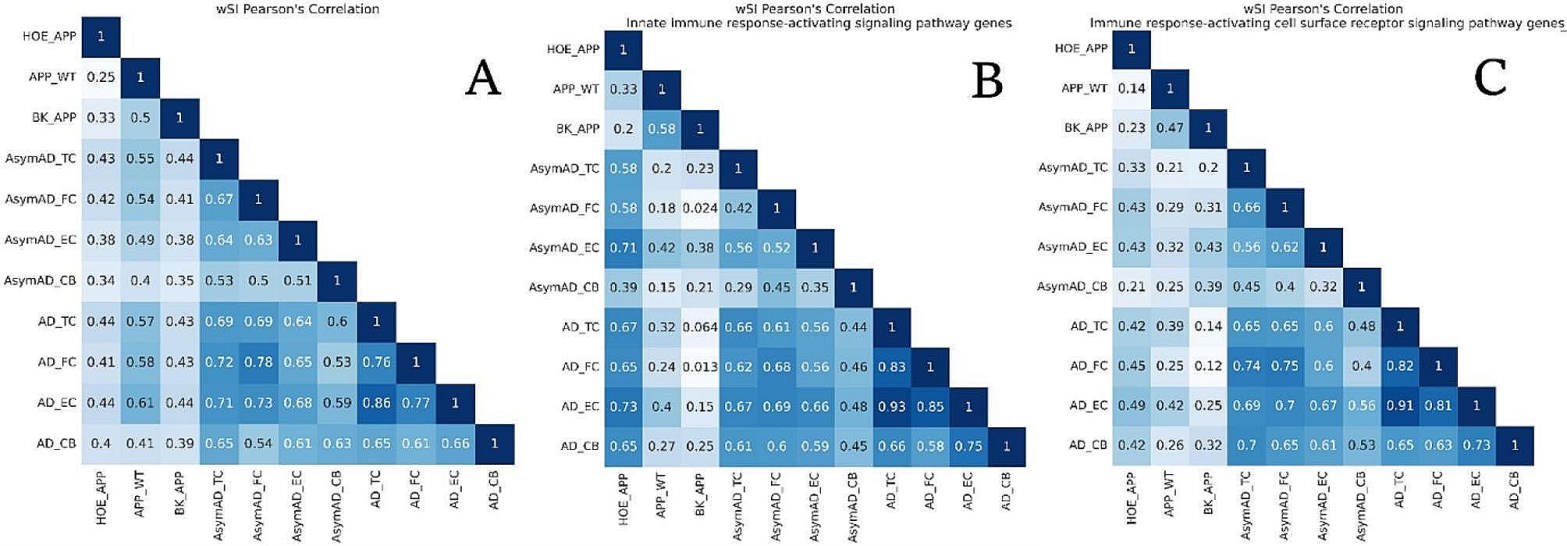
Correlation matrix between *wSI* of the genes. **A**: Correlation matrix of the 4211 genes contained in all groups. **B**: Correlation between *wSI* of the 59 genes belonging to the biological process innate immune response-activating signaling pathway. **C**: Correlation between *wSI* of the 82 genes belonging to the biological process immune response-activating cell surface receptor signaling pathway

**Fig. 10:**
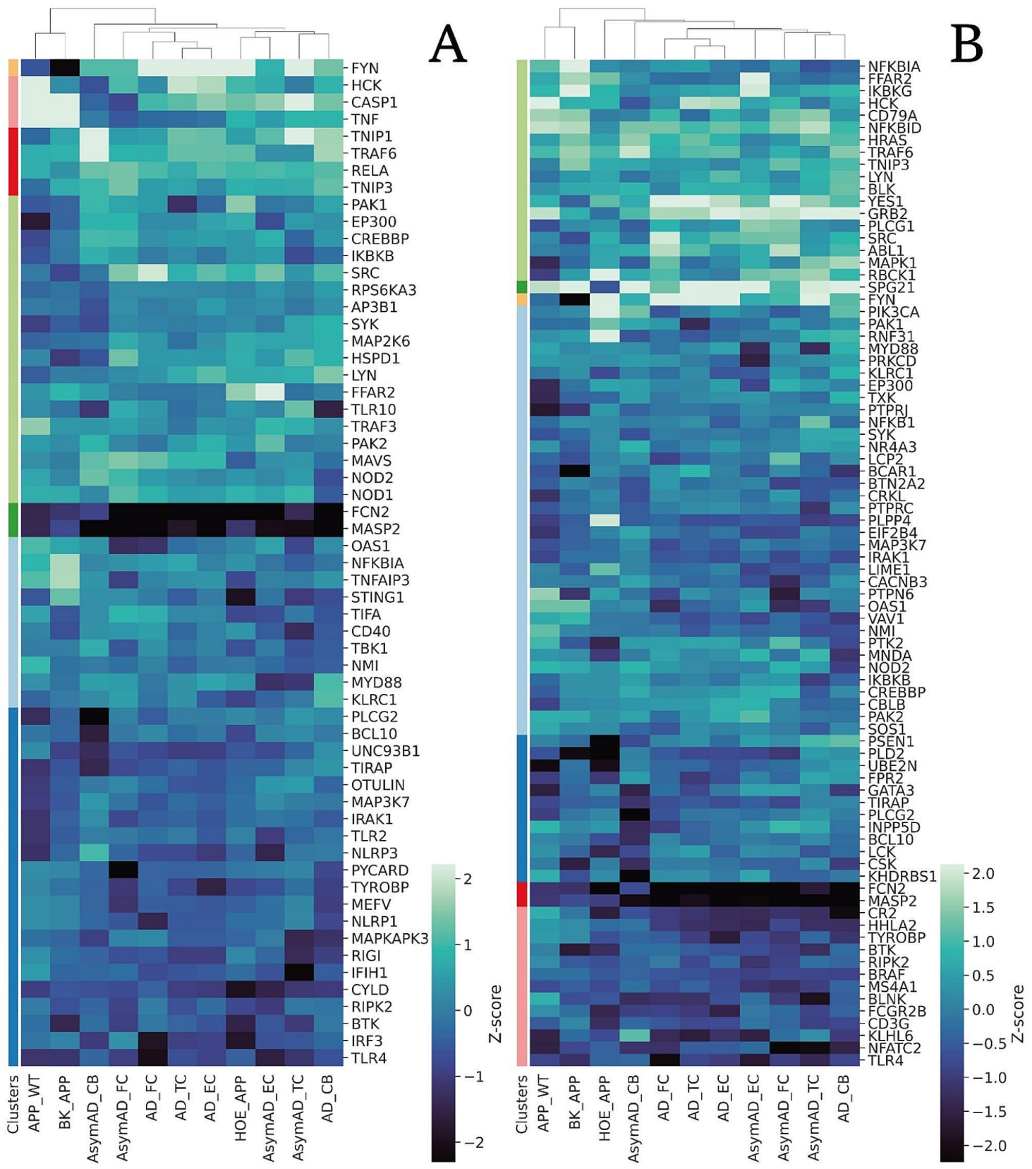
*wSI* clustered heatmap of proteins contained in GO BPs related to immune response. **A ***wSI* of the 59 genes belonging to the biological process innate immune response-activating signaling pathway. **B ***wSI* of the 82 genes belonging to the biological process immune response-activating cell surface receptor signaling pathway

The *wSI* of each of the common proteins among all constructed networks was used to verify the correlation between the groups (Fig. 9 and Fig. S3). This analysis indicates whether the different groups compared have the “dominance” of the genes in the PPI network distributed in a similar way. BK_APP and HOE_APP have a lower to moderate correlation with the human dataset compared to APP_WT that has moderate to high correlations (Fig. 10A). This indicates that, in the topological context of the network, the APP_WT system favors proteins similarly to human disease. On the other hand, treatments with BK or HOE-140 generate a change in the model, mischaracterizing it, of its proximity to human tissues. The correlation of the APP_WT group is always higher for TC, FC, and EC tissues than with CB, which is consistent with the low or null impact of AD on CB. Among the three tissues affected by AD, the one with the highest correlation with APP_WT is EC, one of the first regions affected by the disease. For these same three tissues, the correlation of APP_WT with symptomatic patients is greater than with asymptomatic ones. Indicating that the model more closely represents patients with clinical diagnoses, advanced Braak stage, and neuropathological assessment at autopsy showed hallmark AD pathology.

The 4211 proteins common to all groups enrich, according to the Gene Ontology bank, two biological processes related to the immune response: innate immune response-activating signaling and immune response-activating cell surface receptor signaling pathways. The *wSI* of the genes that enrich these processes was used to construct a correlation matrix between the groups, (Fig. 9B and C). HOE_APP, APP_WT, and BK_APP, in this order, have a decreasing correlation with human tissues. That is, the treatment with HOE-140 alters the model of neurospheres bringing the topological position of the genes of HOE_APP closer to those of human tissues with AD. BK treatment decreases this correlation. This decreasing pattern and the correlations are significantly higher in the innate immune response-activating signaling pathway, indicating a targeted action of HOE-140 in this pathway. For this pathway and in HOE_APP, correlations are higher in symptomatic patients and especially in AD_EC, indicating that this treatment increases the dominance of genes affected by AD.

The genes present in the enriched processes related to the immune system were used to cluster the experimental groups and the tissues of patients with AD (Fig. 10 and clusters in Table S3). In both processes the APP_WT and BK_APP groups are part of the same group and HOE_APP is outside this group. In the innate immune response-activating signaling pathway (Fig. 10A), HOE_APP clusters with AsymAD_EC, AsymAD_TC, and AD_CB. Using the UMAP method, none of the asymptomatic groups of patients with AD were discriminable from healthy patients, (Fig. S4). Thus, the wSI of the genes of this biological process indicates that HOE_APP is closer to groups that are spared by AD (AD_CB) and to groups in which the effect of AD is not sufficient to differentiate patients from healthy controls. In this process, NFKBIA, CD40, MYD88, and IFH1 stand out for presenting significant differences between the groups (AsymAD_CB, AsymAD_FC, AD_FC, AD_TC, AD_EC) and (HOE_APP, AsymAD_EC, AsymAD_TC and AD_CB), p of 0.02, 0.0006, 0.04 and 0.004, respectively. In the immune response-activating cell surface receptor signaling pathway HOE_APP is isolated from all other groups, indicating a treatment-related change in the dominance of genes in this process.

## Discussion

It has long been proposed that neuroinflammation is an important pathological feature related to AD, promoting disease progression [24, 25]. It is considered an inherent host mechanism involved in the protection of the brain against infection and injury, and leads to increased production of several proinflammatory cytokines, chemokines, and inflammatory markers, particularly by microglia and other glial cells [22, 25]. In the present study, we show that the treatment of neurospheres with BK increased the expression of genes related to immune responses, suggesting that an inflammatory state was reinforced. On the other hand, the decrease in the expression of genes related to immune responses by HOE-140 suggests that it was able to attenuate the inflammatory state of APP/PS1 neurospheres. When we specifically analyzed genes, whose expression rates were modified after BK and HOE-140 treatments, we confirmed that the vast majority of them were related to microglia-mediated neuroinflammation, a key player in AD pathogenesis. Importantly, these changes were detected at an early developmental stage, indicating that familial AD has a developmental origin, as already suggested in our previous work [7].

By analyzing changes in gene expression of neurospheres treated with BK and HOE-140, it was possible to determine that many of the genes modulated by the treatments are involved in neuroinflammation mediated by microglia. Here we highlight the described correlations for some of these genes or their respective molecules, which are related to neuroinflammation or AD. For example, in genome-wide association studies, Ly and colleagues (2021) described BCL3, BLNK, LILRB4, and TREM2 as microglial genes involved in AD pathogenesis implicated by AD genomics, and therefore considered genetic risk factors for AD [26]. The molecule CD14 is a known co-receptor of TLR2 and both act together to bind fibrillar beta-amyloid and activate intracellular signaling [26, 27]. The phosphotyrosine phosphoprotein TYROBP (DAP12) is enriched by microglia and forms functional complexes with C3R and TREM2. TREM2 and C1QB are thought to be involved in the switch from homeostatic microglia to disease-associated microglia [28]. Daily and colleagues (2023) described the increased expression of CASP4 by microglia from a mouse model of AD (5xFAD) and considered CASP4 as a regulator of neuroinflammation in AD [29]. EMR1 is the mouse gene encoding F4/80 antigen, adopted as a marker for mouse mononuclear phagocyte cells [30]. The chemokines CCL2, CCL3, CCL4, CCL5, CXCL10, and CXCL5 and the chemokine receptor CX3CR1 orchestrate neuroinflammation by attracting and activating microglia and play an important role in cell-to-cell communication in the CNS [31]. IGSF6 and LY86 have been described as microglial genes identified by TMEM119 expression profile [32]. Tumor necrosis factor (TNF) is an important proinflammatory cytokine that initiates and regulates the cytokine cascade in inflammatory responses and is upregulated in AD patients [32]. Expression of BST2 in microglia is significantly increased during the development of ALS (amyotrophic lateral sclerosis) [33]. GBP5, CX3CR1, and ISG15 are involved in the innate immune response and their relevance to AD neuroinflammation will be discussed later.

The quantification of genes related to inflammatory responses and cell-to-cell communication in the CNS by RT-PCR demonstrated a decreased expression in C3, CCL12 and CCL5 in HOE_APP neurospheres. The chemokines CCL5 and CCL12 may be released by microglia, neurons, and astrocytes and participate in the activation of the immune cells, with CCL5 attracting microglia and CCL12 attracting peripheral blood leukocytes [31]. Popiolek-Barczyk and collaborators (2020) [34] demonstrated that CCL12 is involved in the activation and modulation of the complement cascade by increasing the expression of a component of the classical complement pathway. The same study demonstrated that CCL12 directly influences the activation of microglia and the secretion of immune factors [34]. C3 is the central molecule of the complement cascade, produced by glia surrounding plaques in the AD brain, and contributes to age and plaque-related synapse and neuronal loss [35]. Furthermore, an increase was observed in the expression of TLR2 and CCL12 in BK_APP neurospheres. Toll-like receptors (TLR) 2 are expressed in microglia and are activated by amyloid-$\beta$ (A$\beta$), the main constituent of the amyloid plaques in AD [36].

In APP_WT, only processes with positive mean FCs were enriched. Some of those are related to AD, such as regulation of interferon-β production, regulation of inflammatory responses, upregulation of interleukin-6 production, cellular responses to lipopolysaccharide, microglial cell activation and synapse pruning, with average FCs of 0.95, 1.00, 1.22, 0.90, 1.21 and 2.33, respectively (Fig. 4B). The enrichment of these processes is in accordance with the pathophysiology and representativeness of the APP/PS1 neurospheres model with AD, as already described in the literature [7]. Among the BPs enriched in HOE_APP, the regulation of responses to biotic stimulus and to cytokine stimulus (Fig. 4A) stands out for having negative mean FCs − 0.22 and − 0.29, respectively. The first of them is also enriched in BK_APP with FCs of 0.35 (Fig. 4C), meaning that the proteins from this BP have an average increase in expression in the group treated with BK and a decrease in the group treated with HOE-140. This BP is related to any intensity modulation of processes, such as the innate immune response and the lipopolysaccharide-mediated signaling pathway [37]. Among the BPs with the highest mean FCs in HOE_APP is T cell activation involved in immune response with FCs of 0.34. T cell activation in AD remains a topic of debate. A consensus exists regarding abnormal behaviors of T cells in the progression of AD. However, depending on the activated T-cell subsets there may be pathological or neuroprotective contributions [38]. The processes enriched by BK_APP, such as upregulation of interleukin-1 beta production and cytokine-mediated signaling pathways, hippocampal neuron apoptotic processes, neuroinflammatory responses, microglial cell activation involved in immune response and synapse pruning (Fig. 4C and Table S4) stand out for having positive mean FCs, indicating the impact of BK treatment on neurodegeneration and immune system dysfunction. The only BP in common between HOE_APP and APP_WT is the defense response to viruses. On the other hand, there are 13 common BPs between BK_APP and APP_WT, such as upregulation of interleukin-1 beta production, microglial cell migration, cytokine-mediated signaling pathway and synapse pruning, all with positive mean FCs (Fig. 4B and D and Table S5). The similarity between the processes enriched by BK_APP and APP_WT and the average FCs of each of them indicates the intensification of these BPs generated by BK treatment.

The pathways enriched by the genes in BK_APP have a positive mean FCs and are mostly related to the cell cycle (Fig. 5A). Upregulation of the cell cycle modulator is associated with AD [39]. This and other evidence gave rise to two hypotheses to explain cell cycle dysfunction in AD: senescence of neurons [40] and re-entry of the neuron into the cell cycle [41]. Both are proposed etiological processes of AD and can lead to neurodegeneration via different pathways [42]. Further evidence of the relationship between BK treatment and cell cycle dysfunction is the enrichment of the CC inner kinetochore with an average FCs equal to 0.27 (Fig. 5D). This location is related to the cell cycle as it plays a central role in chromosome segregation [43]. On the other hand, HOE_APP enriched three pathways, all related to the immune system, (Fig. 5B) [44, 45]. All of these pathways have on average upregulated genes, indicating the influence of HOE-140 on the AD model. Among the CCs enriched in APP_WT, T cell receptor complex stands out as it has an average FCs of 2.93 (Fig. 5C). As mentioned previously, the role of T cell activation in AD has an ambiguous interpretation, as it may cause pathogenic or neuroprotective effects [38].

The correlation of gene expression between treatments reveals that ISG15, Ceruloplasmin (CP), GBP5, CX3CR1, and Ras-related C3 botulinum toxin substrate 2 (RAC2) are common between the two species. ISG15 plays a vital role in the innate immune response as a negative regulator of type I interferon [38, 46]. ISG15 has a significant increase in expression between healthy controls and patients with mild cognitive impairment (MCI) and shows a decrease in patients with AD. On the other hand, the type I interferon-stimulated gene family has expression levels positively correlated with the clinical progression of AD [45]. CP is the key copper transport protein in plasma and an iron metabolism regulator, and acts in preventing free radical formation [47]. CP has increased expression during inflammation and infection [48]. In addition, it has a correlation with neurodegenerative diseases such as AD, Parkinson’s disease, and Wilson’s disease, as these disorders exhibit alterations in the metabolism of metal ions, accumulation of iron in the brain, and decreased activity of CP [49]. GBP5 is an interferon-induced GTPase, acts in the innate immune response, and is altered in neurodegenerative systems and models [50]. GBP5 is upregulated in mice microglia exposure to Aβ peptide [51, 52]. CX3CR1 may act on immune response, inflammation, cell adhesion, and chemotaxis [53–56]. CX3CR1 deficiency alters microglial activation and reduces neurodegeneration and amyloid-β deposition in AD models [57]. Finally, RAC2 in an active state binds to a variety of effector proteins and regulates processes such as phagocytosis [58]. RAC2 is overexpressed in AD patients [59] and in the 5×FAD AD mouse model of 4 months age [52].

*wSI* in the HOE_APP, APP_WT, and BK_APP progression shows that EMP1, TRAF1, STING1, SNAP29, STX4, and UBQLN1, have extreme PC values. The first three are PC positive and the others are negative. EMP1 expression is upregulated in the cortex, cerebellum, and in human postmortem brain microglia from AD patients [60, 61]. TRAF1 is differentially expressed and associated with AD and M1 macrophages, the central immune cells in this disease [62]. STING1 begins a cellular innate immune response cascade that is activated in AD patients and aged mice. Besides that, the STING1 inhibitor improved AD pathogenesis in 5×FAD mice [63]. SNAP29 is significantly decreased in AD patients [64]. STX4 has a dual role in AD pathogenesis. In macrophages, STX4 is pro-inflammatory and promotes the exocytosis of chemokines [65]. In oligodendrocytes, in turn, STX4 helps ontrolling the homeostasis of the myelin membrane by vesicle fusion to add proteins and lipids and maintain the normal information flow between neurons [66]. Finally, UBQLN1, when overexpressed in a transgenic mouse, improves the cognitive outcomes in spatial learning and memory performance in both radial arm water maze and Y-maze tests. UBQLN1 overexpressed in APP/PS1 mice decreases amyloid-β accumulation and cognitive and motor deficits caused by AD [67]. In other words, in HOE_APP, APP_WT, and BK_APP, the proteins with increased *wSI* play significant and well-understood roles in the immune system dysfunctions caused by AD, and proteins with decreased *wSI* promote opposite effects.

In comparison with human AD patients, HOE_APP are also notable for having less similarity with the other groups (Fig. 8B). Among the 11 genes, we can highlight DNA excision repair protein ERCC-8, ubiquitin carboxyl-terminal hydrolase 13 and complement C4-B (ERCC8, USP13, and C4B, respectively) for generating this difference. ERCC8 expression is upregulated compared to the other groups, except to AsymAD_CB and AD_CB, which present comparable levels. ERCC8 encodes DNA excision repair complementation (ERC) proteins, and when mutated,  causes the Cockayne syndrome [68]. This syndrome generates accelerated aging of the patient, which in turn is a risk factor for AD [69] and is associated with dysfunctions of the immune system [70]. USP13 is a de-ubiquitinase that controls the protein ubiquitination cycle and is overexpressed in the brains of patients with AD and Parkinson’s disease [71, 72]. USP13 knockdown leads to a reduction of amyloid plaques and hyperphosphorylated tau [73]. However, in all regions of the human dataset and in APP_WT and BK_APP, USP13 expression was downregulated. USP13 is overexpressed only in HOE_APP. Given that the data analyzed in the present work were all generated by transcriptomics, possible post-transcriptional or translational regulation may occur to increase USP13 expression. HOE-140 treatment can alter this regulation, resulting in the observed accumulation of USP13 transcripts. C4B is an opsonin that acts in the classical pathway of the component system promoting immune response and inflammation [74]. C4B has already been identified as highly expressed in AD brains and indicated as an initiator of membrane lysis in neurons and glia during the process of neurodegeneration [75–78]. C4B stands out for being downregulated in its expression levels only in HOE_APP and AD_CB, indicating that the immune response and inflammation are mitigated in these groups. Protein mono-ADP-ribosyltransferase PARP9 (PARP-9) is a gene involved in inflammatory processes, increased cell migration, and macrophage regulation [79]. This gene was downregulated only in the HOE_APP group, indicating the role of HOE-140 in the immune system of the AD model.

## Conclusions

The expression of genes related to immune responses is influenced by treatments applied to neurospheres, reinforcing or reducing the inflammatory state induced by mutations related to AD pathology. BK treatment increases the expression of genes that are involved in microglia-mediated neuroinflammation and that enrich processes, pathways, and cellular locations related to immune impairment, neurodegeneration, and cell cycle, thereby amplifying biological processes already enriched by the genetic burden associated with AD itself. On the other hand, treatment with HOE-140 is capable of promoting the reduction of AD-related changes caused in these systems, suggesting that modulating BK activity in AD might be an important way to slow the progression of the disease. Our results highlight the potential of transcription profiling to reveal the therapeutic potential of candidate therapeutics in the context of AD. Specifically, we show how expression profiling of treatments reveals its disease-interfering potential through its driving gene expression.

### Electronic supplementary material

Below is the link to the electronic supplementary material.

Supplementary Material 1

Supplementary Material 2

Supplementary Material 3

Supplementary Material 4

Supplementary Material 5

Supplementary Material 6

Supplementary Material 7

Supplementary Material 8

Supplementary Material 9

## Data Availability

Raw and normalized microarray data were deposited in the GEO database (https://www.ncbi.nlm.nih.gov/geo) under accession GSE246792.

## Abbreviations

NPCs: Neural progenitor cells
APP: Amyloid precursor protein
PSEN 1: Presenilin 1
PSEN 2: Presenilin 2
AD: Alzheimer’s disease
fAD: Familial Alzheimer’s disease
BK: Bradykinin
CNS: Central nervous system
Mo/HuAPP695swe: Chimeric mouse/human amyloid precursor protein
PS1-dE9: Mutant human presenilin 1
APP/PS1: Double transgenic mice expressing a chimeric mouse/human amyloid precursor protein and a mutant human presenilin 1
CCL12: C-C motif chemokine 12
CCL5: C-C motif chemokine 5
CCL3: C-C motif chemokine 3
C3: Complement C3
CX3CR1: CX3C chemokine receptor 1
TLR2: Toll-like receptor 2
TNF alpha: Tumor necrosis factor alpha
Iba-1: Allograft inflammatory factor 1
Lys10-BK: kallidin
PCR: Polymerase chain reaction
DMEM: Dulbecco’s modified Eagle’s medium
EGF: Pro-epidermal growth factor
FGF2: Fibroblast growth factor 2
APP_WT: APP/PS1’s neurospheres untreated
BK_APP: APP/PS1’s neurospheres treated with 1µM BK
HOE_APP: APP/PS1’s neurospheres treated with 1µM HOE-140
SAM: Significance analysis of microarrays
RT-PCR: Quantitative real-time reverse-transcription polymerase chain reaction
RT: Reverse transcription
FCs: Fold changes
BPs: Biological processes
CCs: Cellular components
GO: Gene Ontology
FDR: False Discovery Rate
HOE_WT: List of genes which the putative FCs is the sum of the FCs of the gene in APP_WT and in HOE_APP
BK_WT: List of genes which the putative FCs is the sum of the FCs in APP_WT and in BK_APP
PC: Pearson correlations
wSI: Weighted system impact
PPI: Protein-protein interaction
EC: Entorhinal cortex
TC: Temporal cortex
FC: Frontal cortex
CB: Cerebellum
AsymAD: Patient which were defined as clinically dementia-free at the time of death, but neuropathological assessment at autopsy showed hallmark AD pathology
BCL3: B-cell lymphoma 3 protein
BLNK: B-cell linker protein
C1QB: Complement C1q subcomponent subunit B
CASP4: Caspase-4
CCL2: C-C motif chemokine 2
CCL4: C-C motif chemokine 4
CD14: Monocyte differentiation antigen CD14
CXCL5: C-X-C motif chemokine 5
EMR1: Adhesion G protein-coupled receptor E1
FAS: Fatty acid synthase
GBP5: Guanylate-binding protein 5
GCH1: GTP cyclohydrolase 1
IFIT1: Interferon-induced protein with tetratricopeptide repeats 1
IGSF6: Immunoglobulin superfamily member 6
ISG15: Ubiquitin-like protein ISG15
LILRB4: Leukocyte immunoglobulin-like receptor subfamily B member 4
TREM2: Triggering receptor expressed on myeloid cells 2
TYROBP: TYRO protein tyrosine kinase-binding protein
VAV1: Proto-oncogene vav
VCAM1: Vascular cell adhesion protein 1
BST2: Bone marrow stromal antigen 2
C4B: Complement C4-B
CXCL10: C-X-C motif chemokine 10
DDX58: Antiviral innate immune response receptor RIG-I
GBP6: Guanylate-binding protein 6
GPR65: Psychosine receptor
IFIH1: Interferon-induced helicase C domain-containing protein 1
IFIT3: Interferon-induced protein with tetratricopeptide repeats 3
LY86: Lymphocyte antigen 86
PTPN6: Tyrosine-protein phosphatase non-receptor type 6
RSAD2: S-adenosylmethionine-dependent nucleotide dehydratase RSAD2
ZC3HAV1: Zinc finger CCCH-type antiviral protein 1
EMP1: Epithelial membrane protein 1
TRAF1: TNF receptor-associated factor 1
STING1: Stimulator of interferon genes protein
SNAP29: Synaptosomal-associated protein 29
STX4: Syntaxin-4
UBQLN1: Ubiquilin-1
DAP12: Phosphotyrosine phosphoprotein TYROBP
CP: Ceruloplasmin
RAC2: Ras-related C3 botulinum toxin substrate 2
ERCC8: DNA excision repair protein ERCC-8
USP13: Ubiquitin carboxyl-terminal hydrolase 13
C4B: Complement C4-B
ERC: DNA excision repair complementation
PARP-9: Protein mono-ADP-ribosyltransferase PARP9
