# Supplementary figures and images for: Bradykinin promotes immune responses in differentiated embryonic neurospheres carrying APPswe and PS1dE9 mutations
Source: Cell Biosci. 2024 Jun 18;14:82. doi: 10.1186/s13578-024-01251-3 (PMC11184896; doi:10.1186/s13578-024-01251-3)

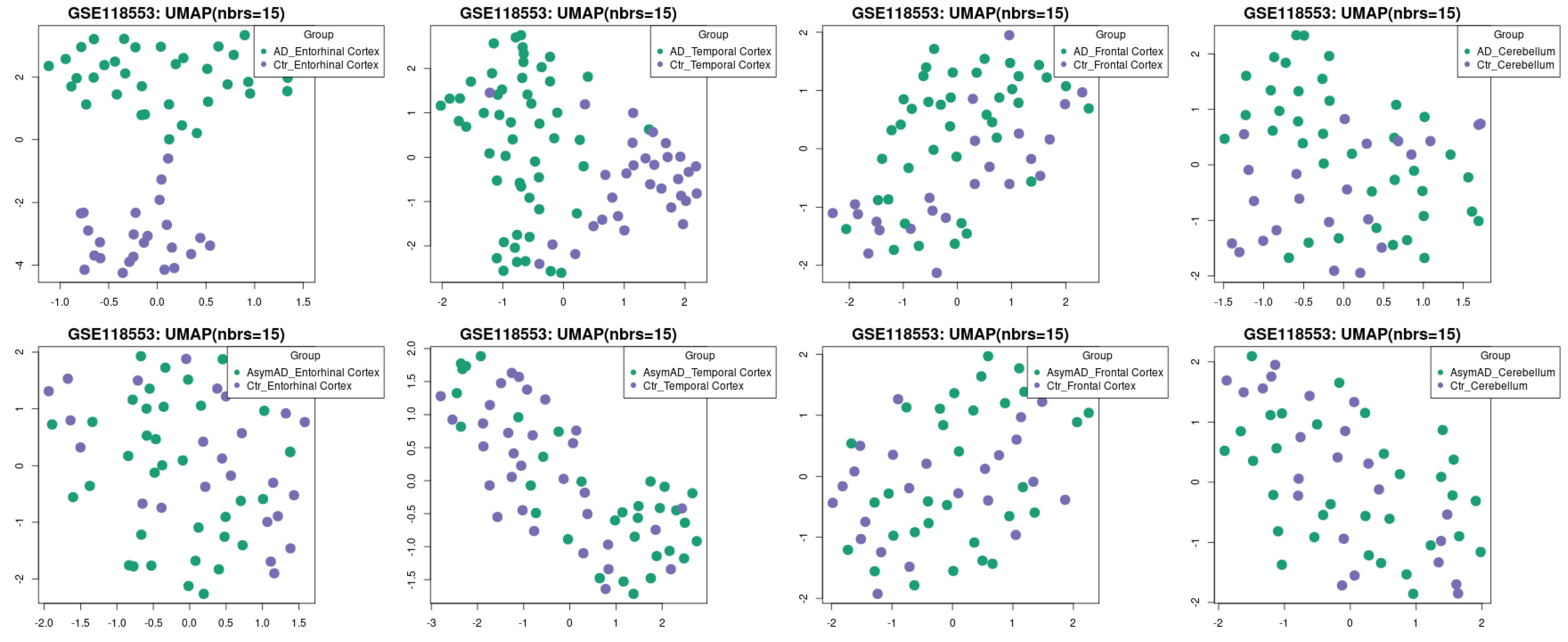

Supplement: Supplementary file 5 — Supplementary Material 5 [file 13578_2024_1251_MOESM5_ESM.png]

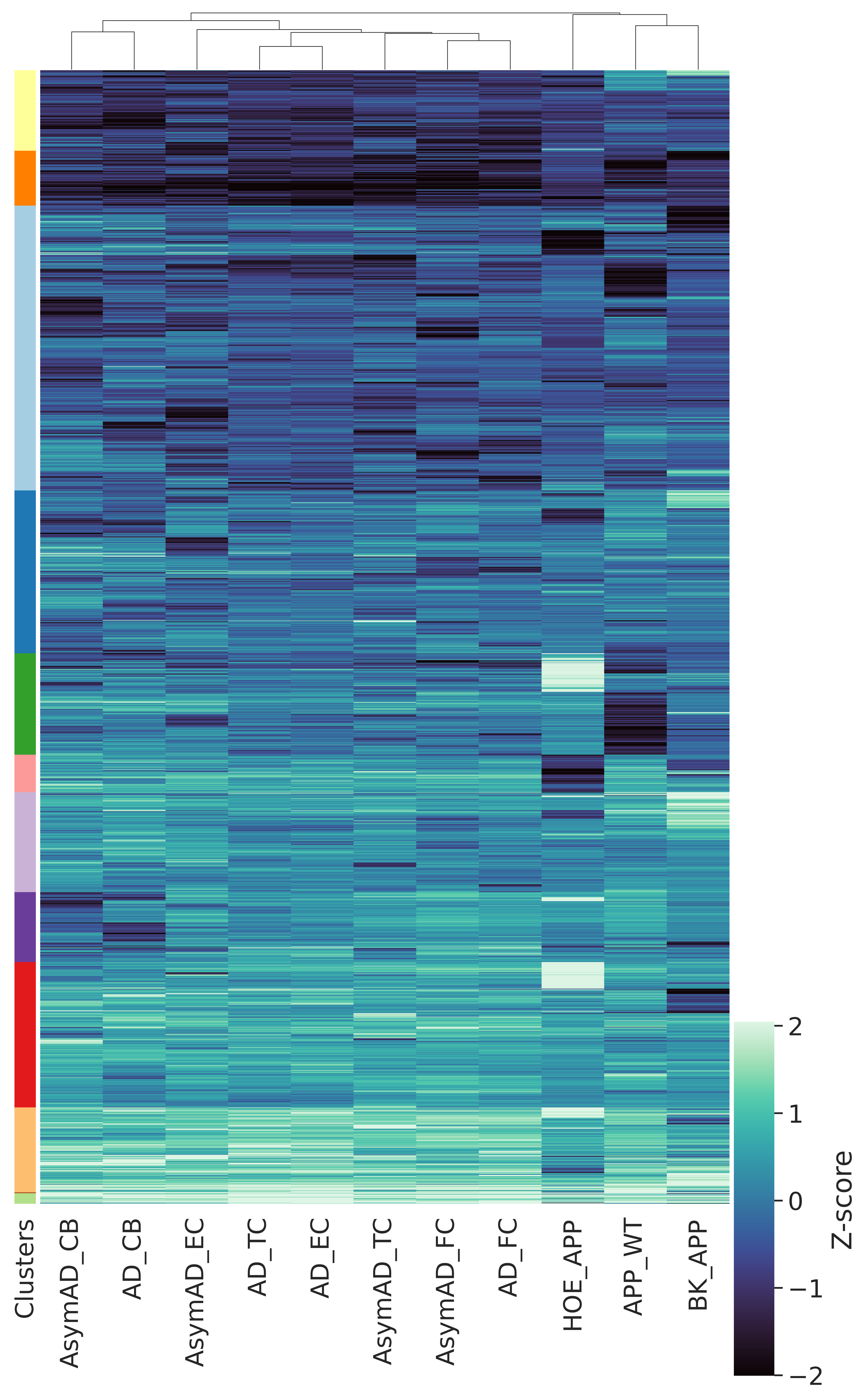

Supplement: Supplementary file 6 — Supplementary Material 6 [file 13578_2024_1251_MOESM6_ESM.png]

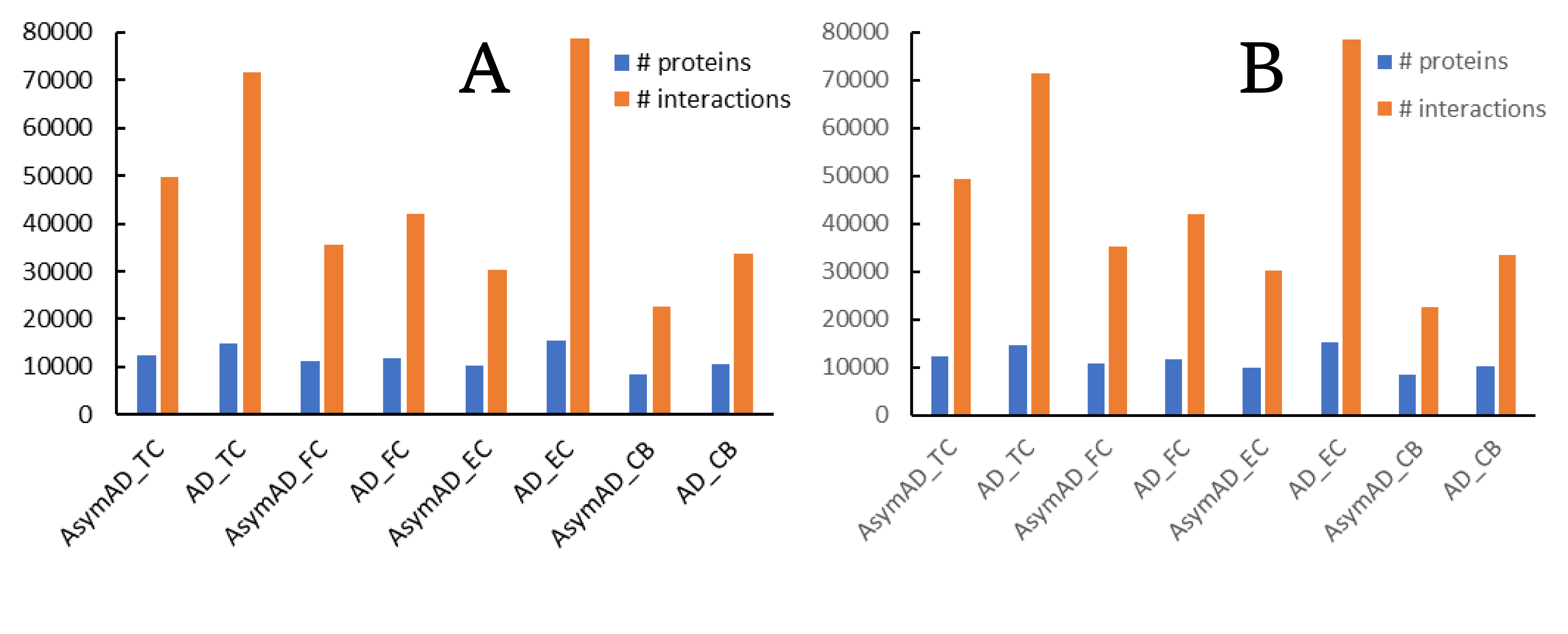

Supplement: Supplementary file 7 — Supplementary Material 7 [file 13578_2024_1251_MOESM7_ESM.png]

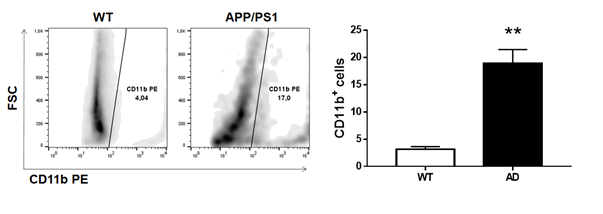

Supplement: Supplementary file 8 — Supplementary Material 8 [file 13578_2024_1251_MOESM8_ESM.png]
