# Supplementary material for: Bradykinin promotes immune responses in differentiated embryonic neurospheres carrying APPswe and PS1dE9 mutations
Source: Cell Biosci. 2024 Jun 18;14:82. doi: 10.1186/s13578-024-01251-3 (PMC11184896; doi:10.1186/s13578-024-01251-3)
Supplement: Supplementary file 9 — Supplementary Material 9 [file 13578_2024_1251_MOESM9_ESM.docx]

**Table S1** Primers of RT-PCR reactions

| **cDNA** | **Primer Forward** | **Primer Reverse** |
| --- | --- | --- |
| **Endogenous PrP** | CCTCTTTGTGACTATGTGGACTGATGTCGG | GTGGAT AACCCCTCCCCCAGCCTAGACC |
| **Tg (APP695)** | GACTGACCACTCGACCAGGTTCTG | CTTGTAAGTTGGATTCTCATATCCG |
| **CCL5** | TGGGGATGCCACTCAGTAAT | ATTTCTTGGGTTTGCTGTGC |
| **CCL12** | GGGAACTTCAGGGGGAAATA | CCATAAACCACTTGGATAAGACG |
| **CCL3** | CACACTGTTTGGTGACAGCTATT | AAAGGGCATATTTATTACTTCTCTGG |
| **C3** | TGCCAGGATCAGAAGTACCA | TAGAGGGCTGGGCTGTAGTC |
| **CX3CR1** | TCCAGTTCTGCAACTTGCTTT | TGGGACCTCTGTAGGAGCAG |
| **TLR2** | CCAAGACCTACCTGGAGTGG | AGGAACTGGGTGGAGAACCT |
| **TNF-alpha** | CACAAGATGCTGGGACAGTG | CATTCGAGGCTCCAGTGAAT |
| **AIF1/Iba** | CCTGATTGGAGGTGGATGTC | GTCTGACTCTGGCTCACGACT |
| **GAPDH** | GCACAGTCAAGGCCGAGAAT | GCCTTCTCCATGGTGGTGAA |

**Table S2** Clusters of genes formed in the heatmap of Figure 8.

**Table S3** Clusters of genes formed in the heatmap of Figure 10.

**Table S4** Whole BP enrichment of BK_APP by GO.

**Table S5** Whole BP enrichment of APP_WT by GO.

**Fig. S1** Flow cytometry analysis of activated microglial cells (CD11b) of differentiated neurospheres from WT and AD samples. The data shown are representative of at least three independent experiments. ** p < 0.01.

**Fig. S2** Number of proteins and interactions in each PPI network. **A** Whole networks and **B** only the main component of each network.

**Fig. S3** Heatmap between *wSI* of the 4211 genes in the analyzed microarray and the human dataset.

**Fig. S4** UMAP of transcriptomes from patients. Includes symptomatic AD, asymptomatic AD, and control group for entorhinal cortex (EC), frontal cortex (FC), temporal cortex (TC), and cerebellum (CB).
